# ‘Telling them “that’s what it says in the guidance” didn’t feel good enough’: moral distress during the pandemic in UK public health professionals

**DOI:** 10.1093/pubmed/fdad220

**Published:** 2023-11-29

**Authors:** Steven M A Bow, Peter Schröder-Bäck, Dominic Norcliffe-Brown, James Wilson, Farhang Tahzib

**Affiliations:** Department of Philosophy, University College London, Gower Street, London WC1E 6BT, UK; Institute for Ethics and History, University of Applied Sciences for Police and Public Administration in North Rhine-Westphalia (HSPV NRW), 52068 Aachen, Germany; Medical Ethics, British Medical Association, London WC1H 9JP, UK; Department of Philosophy, University College London, London WC1E 6BT, UK; Faculty of Public Health, London NW1 4LB, UK

**Keywords:** ethics, mental health, work environment

## Abstract

**Background:**

The study aimed to identify the causes of moral distress in public health professionals associated with the COVID-19 pandemic, and the potential ways of avoiding or mitigating the distress.

**Methods:**

The survey was distributed to all members of the UK Faculty of Public Health between 14 December 2021 and 23 February 2022. Conventional qualitative content analysis was conducted to explore the *situations* in which moral distress arises, the *moral judgments* that led to distress and the *proposed ways to address* moral distress.

**Results:**

A total of 629 responses were received from respondents broadly representative of the public health professional workforce. The main situations causing moral distress were national policy, guidance and law; public health advice; and workplace environments. Moral distress was precipitated by judgments about having caused injury, being unable to do good, dishonest communications and unjust prioritization. The need to improve guidance, communication and preparedness was recognized, though there was disagreement over how to achieve this. There were consistent calls for more subsidiarity, moral development and support and freedom to voice concerns.

**Conclusions:**

The causes of moral distress in public health are distinct from other healthcare professions. Important proposals for addressing moral distress associated with the COVID-19 pandemic have been voiced by public health professionals themselves.

## Introduction

The wake of the COVID-19 pandemic brought international reports^1–4^ of public health professionals experiencing moral distress and injury. In the spring of 2020, the UK experienced a surge in infections, and associated hospitalizations and deaths, which precipitated a nationwide ‘lock-down’ (a restriction on movement outside the home) lasting several months. There was a subsequent significant peak of infections in the winter of 2021, with a range of national and local restrictions and activities in response. It was not till February 2022 that the UK government published its ‘Living with COVID-19’ guidance which marked an end to the most significant aspects of the pandemic response.^5^ As well as enduring the same consequences as the rest of society, public health professionals played a significant role in the pandemic response, and many were directly affected by the major organizational restructure precipitated when the national public health agency was abolished mid-pandemic.^6^

Moral distress can be defined as psychological distress arising from a ‘moral event’ (an experience to which the subject attaches a significant moral judgment),^7^ and can lead to moral injury, i.e. subsequent long-term psychological and/or physical symptoms.^8^ Not only can moral distress and injury harm the professionals who suffer them, but the cumulative disruption to the workforce risks harm to the public they serve. Moreover, incidents of moral distress may indicate the occurrence of some injustice which requires redressing. However, there is limited evidence on how to reduce or address moral distress and injury.^9–11^

Past surveys on the causes of moral distress focus on the wider UK medical workforce.^1^^,^^12^^,^^13^ However, medical doctors account for no more than half of the number of UK public health consultants,^14^ and public health doctors only constitute a small proportion of UK medics (E.g. 2.1% of the respondents to the BMA survey were public health specialists.). Only two studies explored moral distress in public health sub-specialities. Sunderland *et al.* identified moral distress associated with a perceived lack of political will for ‘upstream’ health promotion in Australian and Canadian health promotion practitioners,^15^ and Cooke *et al.* identified moral distress associated with excessive work-related stress, unsupportive, ineffective or corrupt leaders, inadequate resources, and poor access to public health services in field epidemiologists from 54 countries.^16^ However, neither study explored ways to address the identified moral distress, and, to date, no studies have looked at the public health professional workforce as a whole, or at the impact of the COVID-19 pandemic.

To address this gap, the UK public health professional membership body, the Faculty of Public Health (FPH) surveyed its members about experiences of moral distress since the start of the COVID-19 pandemic, to explore (i) what were the situations in which moral distress arose? (ii) what were the moral judgments that led to distress? and (iii) what ways of addressing moral distress were proposed?

## Method

### Design

An electronic cross-sectional survey of the members of the UK Faculty of Public Health (c. 4000),^17^ distributed via email between 14 December 2021 and 23 February 2022.

### Measures

The survey collected structured information about experiences of moral distress and injury, demographics, professional background and ethical training and education, and open-ended questions about the situation that caused distress, and what would help avoid or mitigate such situations (see Supplementary Materials for survey questionnaire).^4^

There remains debate over the definition of moral distress, with many authorities arguing that the paradigmatic definition, i.e. distress precipitated by a subject being obliged to act in a way they consider to be morally wrong due to institutional constraints, is unnecessarily narrow.^7^^,^^8^^,^^18–23^ In this survey, we asked participants about distress caused by four types of moral event, namely where:

(i) they had to do something that they thought was ethically problematic (morally wrong)
(ii) they did something that they thought was the ethical (morally right) thing
(iii) they had to do something where they were not sure what the ethical (morally right) thing to do was
(iv) their colleague(s), or organization, did something that they thought was ethically problematic (morally wrong)

These types could be termed ‘paradigmatic moral distress’, ‘moral criticism’, ‘moral uncertainty’, and ‘moral outrage’, respectively. This broad range of definitions of moral distress was chosen to avoid excluding salient experiences, with the view to distinguishing between narrower types of moral distress in the analysis stage.

### Analysis

Conventional qualitative content analysis applied to the free-text survey responses.^24^ Two researchers worked independently, using Nvivo software, each maintaining an activity and reflective log. Based on the first 25 responses, they developed independent coding schemes for each free-text survey item about moral distress and its prevention. The researchers then reviewed schemes together, deciding to merge data across survey items (due to conceptual overlap), and group codes against the three research questions. The researchers encoded the remaining responses, refining their independent code lists as they went, until reaching saturation (determined when additional responses no longer affected the code list) independently (at ~50% of sample). Codes were then categorized independently, then compared, defined and reconciled between researchers. Finally, the researchers independently grouped their codes into general themes (highest level categories), before reconciling the two sets together to agree a set of themes that reflected the data (Table 1).

**Table 1:** Themes and sub-themes identified from survey responses

**#**	**Theme**	**Sub-theme(s)**
**Situations in which moral distress arises**		
1A	Developing/implementing national policy, guidance and law.	
1B	Giving public health advice.	
1C	Workplace relationships and environments.	
**Moral judgment that led to distress**		
2A	Causing injury to others.	Pursuit of public health goals at the expense of other goods. Doing harm through blind obedience to guidance.
2B	Inaction/inability to do good.	
2C	(Dis)honesty in public health messaging.	
2D	Unjust prioritization decisions.	Advising without sufficient resources.
**Proposed ways to address moral distress**		
3A	Better decision-making.	Better national policy and guidance. Subsidiarity/decentralization of decision making. Flexibility in national guidance
3B	Communication and participation.	
3C	Moral development and personal support for practitioners.	
3D	Pandemic planning, preparedness and resourcing.	
3E	Freedom to voice concerns and conscientious objection.	
3F	Acceptance/no mitigation possible.	

## Results

Overall, 629 survey responses were received, which equates to ~16% of the FPH membership. Demographics suggested respondents were broadly representative of the UK public health professional workforce (see supplementary information for demographic breakdown).^14^ Of the 405 (64%) who reported one or more experience of moral distress associated with their own action (or inaction) since the start of the pandemic, 300 (74%) responded to one or more of the open-ended questions about the cause or possible mitigations of the moral distress they had experienced. Thirteen themes and six sub-themes were identified against the three research questions (summarized in Table 1 and detailed below).

### Situations in which moral distress arises

#### Developing/implementing national policy, guidance and law

Multiple reports of moral distress related to concerns over the content of (predominantly COVID-19) national policy, guidance and law which they were required to implement. Some, especially those involved in developing COVID-19 policy, guidance and law, felt distressed over the perception that politicians had a stronger voice than public health specialists in the development process.

#### Giving public health advice

Multiple respondents described situations in which the public health advice they provided was challenged or ill-received by its recipients, when, e.g. giving public interviews, providing outbreak management advice or conducting contact-tracing. In some cases, the distress was linked to conflict with, or aggressive criticism by, the recipient. In others, it was caused by the respondents’ strong sympathetic response to the emotional reaction, or perceived suffering, of the recipient of advice.

#### Workplace relationships and environments

Multiple reports of moral distress, particularly in senior roles, related to aspects of the respondent’s working environment—including co-worker relationships, workload and pressure from external institutions. Some felt undermined or bullied at work. Others experienced a moral conflict between delivering their professional obligations fully (including doing large amounts of overtime) and maintaining their own personal health or wellbeing.

### Moral judgments that led to distress

#### Causing injury to others

A major cause of moral distress was the perception of having done harm to others through, e.g. implementation of policies or giving public health advice—especially where the pursuit of the physical health of the population (especially via COVID-19 measures) was at the expense of other goods (e.g. social, mental and financial wellbeing). Examples included preventing relatives visiting dying care home residents, advising against the religious ritual washing of a deceased body, and advising a school to send home a symptomatic child who said to teachers:

‘Daddy will hit me if I make him take time off work.’

Conversely, some respondents thought that other social goods were given *too much weight* at the expense of COVID-19 measures. Sometimes these decisions were seen as a *moral dilemma*, with no obviously ‘right’ way of resolving the conflict.

Some reported that distress arose from feeling obliged to obey or propagate COVID-19 guidance and rules that were considered to be erroneous, inadequate or inappropriate to the situation at hand (or feeling conscientiously bound to depart from guidance, with an attendant risk of professional and legal consequences). Examples included advising care homes to accept patients discharged from hospital without a negative test, and advising people with a recent history of COVID-19 infection to self-isolate again. One participant summed this up as follows:

‘(Telling them) “that's what it says in the guidance” didn't feel good enough.’

#### Inaction/inability to do good

Multiple respondents, especially local authority Directors of Public Health (DPHs), reported distress from failing or delaying to take positive action, due to external constraints. Examples included feeling unable to influence the content or execution of guidance, being turned down when offering to support the COVID-19 response, and the opportunity costs of prioritizing acute COVID-19 work over other activities.

#### (Dis)honesty in public health messaging

Some respondents reported moral distress after feeling pressured into presenting information in a dishonest or misleading way—where they felt that information was false, against their own appraisal of evidence, and/or politically motivated. One respondent stated plainly:

‘(I had) to give out information I knew was wrong.’

Examples included disagreement over the content or interpretation of statistics, and exaggerating expected benefits or downplaying the adverse effects of interventions e.g. vaccination and lockdowns.

#### Unjust prioritization decisions

Moral distress also arose from limitations in resources (e.g. staff, finances, personal protective equipment [PPE], testing capacity, vaccine doses). Sometimes the perceived limitation prevented some good being done or led to increased workload. Sometimes it necessitated difficult decisions about prioritizing between individuals or population groups—which sometimes felt unjust. This caused particular distress when individuals felt that their role meant they were ‘complicit’ in the decision, and even when the decision was constrained by prioritization criteria (e.g. stipulated in national guidance).

### Proposed ways to address moral distress

#### Better decision-making

Multiple respondents suggested moral distress could have been mitigated by improving decision-making, calling for evidence-led policy-making, more input from experts and for more recognition of ethical considerations. Some called for improvements to national (mostly COVID-19) policy and guidance, including development processes, responsiveness to new evidence and the changing context, and accessibility and user-friendliness.

*Subsidiarity*, i.e. situating decision-making responsibility where it can most effectively enable institutions and citizens to pursue their proper goals, was a major theme.^25^ Multiple respondents felt that decision-making should have been decentralized to a greater degree—although a few called for the opposite, i.e. stronger centralization and more nationwide imposition of rules.

Some proposed devolving national powers to regional and local authorities (especially to local DPHs), or to public health professionals and other intermediate institutions, e.g. employers, and the voluntary sector—while recognizing the need for ‘appropriate checks and balances… in place to avoid “maverick” behaviours’. A more modest suggestion, usually from more junior staff, was that national guidance should be more flexible, with scope for local, institutional or professional discretion, explicitly written into it. One called for:

“…more flexibility in guidance for care settings to make their own risk assessments based on individual circumstances. I perceived much of the guidance as cruel and unbalanced.”

#### Communication and participation

Multiple respondents thought moral distress could be reduced by increasing meaningful participation in policy development and decision-making, and improving communication between individuals (e.g. juniors and seniors, politicians and officials) and institutions (e.g. government departments, local authorities and public, private and voluntary sectors). Proposals included specific measures—e.g. rapid feedback mechanisms on guidance implementation, advance warning of imminent policy changes, and seeking views of stakeholders to anticipate and avoid harms to population sub-groups.

#### Moral development and personal support for practitioners

Respondents in all but the most senior roles recommended provision of support for public health practitioners. Suggested measures included promoting a better work-life balance and greater formal mechanisms for emotional support. It was recognized that discussing challenges with colleagues, peers and friends can be instrumental in relieving distress (moral or otherwise), and responding constructively to it. The need for greater awareness of ethical duties, space for ethical reflection and efforts to develop the moral virtues (at both an individual and institutional level) was recognized. Education on morality, ethics and law was often proposed.

#### Pandemic planning, preparedness and resourcing

Senior respondents highlighted the role of pandemic planning and management in mitigating moral distress. Common recommendations included the implementation of learning from previous pandemics and exercises, and maintaining stockpiles of pandemic response supplies, e.g. PPE and vaccines. Multiple respondents saw the solution to moral distress in financial terms, calling for more resources to be directed to, variously, healthcare infrastructure, social services, public health workers, vulnerable groups (via e.g. welfare payments), or ‘the system’ in general.

#### Freedom to voice concerns and conscientious objection

A number of proposals from less senior respondents centered around the freedom to debate and challenge policies without fear of punishment. Rather than explicit external pressure or actual punishment, most of the comments represented concerns about the *perceived consequences risked* if they spoke out (e.g. fear of losing their job). For example, one said:

‘There was… an unspoken pressure to not voice disagreement.’

Respondents suggested this necessitated cultural change, structures that promote debate and protections for conscientious objection and whistleblowing.

#### Acceptance/no mitigation possible

Some saw acceptance—either of moral distress itself, or of the situations in which they may arise—as an effective response, considering such experiences to be an inevitable part of their job. Similarly, multiple respondents simply considered moral distress to be a healthy sign of reflective professional practice.

## Discussion

### Main finding of this study

*Workplace relationships and environments* (1C) and *giving public health advice* (1B) emerged as key situations in which moral distress arose. The latter was a significant part of many public health professionals’ jobs during the pandemic, and may be analogous to the *clinician-patient encounter*—a major focus of moral distress research in the healthcare context.^26^  *National policy, guidance and law* was at the root of much moral distress (1A), which suggests this was an important external constraint experienced by public health professionals during the pandemic, where the more prominent duty of obedience was to institutional instructions, rather than to those of a medical superior.

*Blind obedience to guidance* was another major driver of this moral distress (2Aii), which reveals that many public health professionals (reassuringly) have an underlying sense of right and wrong, or objective ethical standard, which they (less reassuringly) perceived to be at odds with the guidance.

Our research identified the challenges of *achieving justice in prioritization decisions* as an important and distinct cause of moral distress (2D). Prioritizing one service or group is always at the expense of another, and thus raises questions of justice—a well-explored, and hotly contested issue in the bioethics literature.^27^ Public health professionals’ decisions frequently affect whole groups or populations, so any injustice may be felt more keenly compared to clinicians, whose prioritization decisions are usually confined to individual patients.

### What is already known on this topic

Moral distress associated with *caused injury to others* (2A) has parallel themes in healthcare research, such as *treatment causing unnecessary suffering* and *negligence*.^26^ Our finding that *conflicts between the pursuit of health and the pursuit of other goods* (2Ai) were a major driver of this distress, echoes a subtheme of Cooke *et al*.^16^ This kind of moral conflict may be difficult to avoid, since its resolution will often necessarily fall beyond the remit of public health staff, who are professionally bound to prioritize the pursuit of health.

Being *constrained into inaction* (2B), as a cause of moral distress, accords with previous studies in public health and healthcare professionals.^15^^,^^16^^,^^26^ This is a potentially unlimited source of moral distress, as there is always more good that could be done, and limits on a public health professional’s ability to do it.

The moral distress associated with *dishonest public health communication* (2C) may parallel the moral distress felt by clinicians when *withholding information*, *giving false hope* and *covering up errors or ethical violations*.^26^ The *inability to be transparent* was a theme Cooke *et al.* found associated with moral distress in epidemiologists.^16^ Our results replicated this, and suggest that it is not just the withholding of information, but the active communication of untruthful or misleading information, that can cause moral distress in the public health profession at large. This could be a problem inherent to public health communication, wherever its ultimate goal is to change people’s behavior, as there could be a temptation to simplify or exaggerate the facts in order to achieve that goal.

### What this study adds

Uniquely, our study asked respondents for their proposals for addressing and mitigating the moral distress they had experienced. This is of value both in terms of idea-generation and insofar as the respondents are well-placed to assess the options available in concrete situations. This does not mean the proposals should be embraced uncritically. Here we assess the plausibility that they would reduce moral distress and/or injury, the feasibility of implementation and the acceptability of the action required and any attendant consequences.

Several themes represented superficial agreement about the need to make improvements—e.g. to guidance [3Ai], communication [3B] and resourcing [3D], however there was substantial disagreement over the detail. Many proposed methods of ‘improving’ the situation were potentially in competition with each other, or even mutually incompatible, e.g. some respondents called for guidance to be altered *less frequently*, and others *more frequently* etc*.* In such examples, acting on one proposal—to reduce moral distress and injury in one area or for one type of professional—risks increasing the chance of it in another.

However, in other themes, respondents were much more unanimous about the specific action required, making them more conducive for policy adoption. The calls for greater *subsidiarity and decentralization of decision-making* (3Aii) and *flexibility in guidance* (3Aiii) represented a consistent and mutually reinforcing message. The principle of subsidiarity is, at least, partially reflected in the arrangement of the UK public health system,^28^ although our findings suggest this could go further—particularly in light of the pandemic experience, where many decisions were centrally imposed.

Similarly, the consistent call for *freedom to voice concerns and conscientiously object* (3E), echoing the BMA’s call for a more ‘open and sharing workplace culture’ (p15),^1^ seems to offer a plausible solution to moral distress, through encouraging feedback and reflection on ethically questionable decisions, and removing actors from morally problematic situations, respectively.

The call for *moral development and personal support for practitioners* (3C) was unequivocal. It seems plausible that equipping public health professionals to recognize, analyze and reflect on moral questions, and to process attendant emotions, both individually and with colleagues, could avoid or mitigate much moral distress—as well as potentially improving the quality of decision-making.^29^

### Limitations of this study

Although we asked about four different types of moral distress, with the intention of distinguishing between the differential themes arising, many respondents did not strictly observe the intended distinctions, so the items were aggregated in the analysis. This meant that our results pertain to a broader definition of moral distress than many previous studies, which may affect the validity of comparisons. Many responses lacked explicit moral reasoning, which prevented encoding against this research question.

Many proposed interventions for moral distress have the potential to backfire—e.g. devolving power to practitioners could increase moral distress if they are unequipped to handle greater responsibility; training practitioners to recognize moral dilemmas could lower their threshold for experiencing moral distress.^11^ Therefore, proposed solutions should be piloted or subjected to further research to test their effectiveness, feasibility and acceptability.

As not all FPH members responded to the survey, the results may not be representative of the views of all. The intentional focus on the UK workforce, and on experiences associated with the COVID-19 pandemic, means findings may not generalize geographically or temporally.

## Conclusions

This study could support the development of a typology of causes of moral distress in public health professionals, itself a prerequisite for developing a valid measurement tool for prospective research. The causes of moral distress identified in the public health professional workforce bore some correspondence to those dominant in the healthcare literature and the few existing studies in public health, although we identified a number of themes not previously reported. Uniquely, this study provides potentially actionable proposals for addressing moral distress, as identified and articulated by those who have suffered it since the start of the COVID-19 pandemic.

## Supplementary Material

supplementary_materials_fdad220

## Data Availability

Aggregate survey data is provided in Bow et al. (submitted).^4^ Record-level data cannot be shared, due to local information governance and data protection regulations.

## Notes

**Steven M.A. Bow,** Public Health Consultant & Visiting Research Fellow

**Peter Schröder-Bäck,** Professor of Ethics and Sociology

**Dominic Norcliffe-Brown,** Senior Ethics Advisor

**James Wilson,** Professor of Philosophy

**Farhang Tahzib,** Public Health Physician & Chair of Ethics Committee
