# Supplementary material for: ‘Telling them “that’s what it says in the guidance” didn’t feel good enough’: moral distress during the pandemic in UK public health professionals
Source: J Public Health (Oxf). 2023 Nov 29;46(1):194–201. doi: 10.1093/pubmed/fdad220 (PMC10901263; doi:10.1093/pubmed/fdad220)
Supplement: supplementary_materials_fdad220 [file supplementary_materials_fdad220.docx]

‘Telling them “that's what it says in the guidance” didn't feel good enough’: Moral distress during the pandemic in UK public health professionals

# Supplementary information

[Supplementary information 1](#_Toc149333225)

[Supplementary Table 1. Demographic and professional characteristics of respondents 2](#_Toc149333226)

[Survey questions 4](#_Toc149333227)

## Supplementary Table 1. Demographic and professional characteristics of respondents

| **Characteristic** | **N** | **P** |
| --- | --- | --- |
| **Gender** |  |  |
| Female | 308 | 49.0 |
| Male | 148 | 23.5 |
| Other (please specify) | 3 | 0.5 |
| Prefer not to say | 16 | 2.5 |
| Not stated | 154 | 24.5 |
| **Age** |  |  |
| Under 18 | 0 | 0.0 |
| 18-24 | 0 | 0.0 |
| 25-34 | 55 | 8.7 |
| 35-44 | 115 | 18.3 |
| 45-54 | 112 | 17.8 |
| 55-64 | 104 | 16.5 |
| 65+ | 67 | 10.7 |
| Prefer not to say | 22 | 3.5 |
| Not stated | 154 | 24.5 |
| **Disability** |  |  |
| Yes | 49 | 7.8 |
| No | 408 | 64.9 |
| Prefer not to say | 18 | 2.9 |
| Not stated | 154 | 24.5 |
| **Religion** |  |  |
| No religion | 231 | 36.7 |
| Christian – Church of England/Ireland/Wales/Scotland | 82 | 13.0 |
| Christian – Catholic | 37 | 5.9 |
| Other Christian, please describe below | 26 | 4.1 |
| Muslim | 14 | 2.2 |
| Other, please describe below | 9 | 1.4 |
| Hindu | 7 | 1.1 |
| Jewish | 7 | 1.1 |
| Buddhist | 4 | 0.6 |
| Christian – Orthodox | 3 | 0.5 |
| Sikh | 0 | 0.0 |
| Prefer not to say | 54 | 8.6 |
| Not stated | 155 | 24.6 |
| **Ethnicity** |  |  |
| White - Scottish/English/Welsh/Northern Irish/British | 320 | 50.9 |
| White - Any other White background, please describe below | 38 | 6.0 |
| White - Irish | 17 | 2.7 |
| Black - African | 16 | 2.5 |
| Asian - Indian | 12 | 1.9 |
| Asian - Any other Asian background, please describe below | 8 | 1.3 |
| Mixed/Multiple - White and Asian | 6 | 1.0 |
| Asian - Pakistani | 5 | 0.8 |
| Mixed/Multiple - White and Black African | 5 | 0.8 |
| Asian - Chinese | 4 | 0.6 |
| Other - Arab | 4 | 0.6 |
| Mixed/Multiple - White and Black Caribbean | 3 | 0.5 |
| Other - Any other ethnic group, please describe below | 3 | 0.5 |
| Asian - Bangladeshi | 1 | 0.2 |
| Black - Any other Black/African/Caribbean background, please describe below | 1 | 0.2 |
| Black - Caribbean | 1 | 0.2 |
| Mixed/Multiple - Any other Mixed/Multiple ethnic background, please describe below | 1 | 0.2 |
| White - Gypsy or Irish Traveller | 0 | 0.0 |
| Prefer not to say | 27 | 4.3 |
| Not stated | 157 | 25.0 |
| **Country** |  |  |
| England | 479 | 76.2 |
| Outside the UK (please specify) | 63 | 10.0 |
| Scotland | 42 | 6.7 |
| Wales | 29 | 4.6 |
| Northern Ireland | 16 | 2.5 |
| Not stated | 0 | 0.0 |
| **Employer** |  |  |
| Local authority / local government | 206 | 32.8 |
| National public health agency (for example, Public Health England/UK Health Security Agency, Public Health Wales, Public Health Scotland, Public Health Agency N. Ireland) | 140 | 22.3 |
| University or academic institution | 76 | 12.1 |
| Other (please specify) | 64 | 10.2 |
| NHS Trust or Health Board | 53 | 8.4 |
| Department for Health and Social Care / other national government department | 22 | 3.5 |
| NHS England and NHS Improvement | 14 | 2.2 |
| Non-profit organisation/NGO | 12 | 1.9 |
| Private practice | 10 | 1.6 |
| Office for Health Improvement and Disparities (UK) | 5 | 0.8 |
| Not applicable | 26 | 4.1 |
| Not stated | 1 | 0.2 |
| **Job role** |  |  |
| Consultant or Specialist in Public Health | 207 | 32.9 |
| Public Health Trainee / Specialty Registrar | 132 | 21.0 |
| Other (please specify) | 84 | 13.4 |
| Public Health Academic | 59 | 9.4 |
| Director of Public Health | 49 | 7.8 |
| Retired | 39 | 6.2 |
| Public Health Practitioner | 30 | 4.8 |
| Public health manager | 23 | 3.7 |
| Public health nurse or midwife | 4 | 0.6 |
| Not stated | 2 | 0.3 |
| **Duration of professional public health role** |  |  |
| 5 years or less | 147 | 23.4 |
| 6 to 10 years | 112 | 17.8 |
| 11 to 15 years | 80 | 12.7 |
| 16 to 20 years | 78 | 12.4 |
| More than 20 years | 212 | 33.7 |
| Not stated | 0 | 0.0 |
| **Clinically trained** |  |  |
| Yes | 369 | 58.7 |
| No | 259 | 41.2 |
| Not stated | 1 | 0.2 |

## Survey questions

**Question 1. Where have you primarily worked, since the beginning of the pandemic?**

- England
- Scotland
- Wales
- Northern Ireland
- Outside the UK (please specify)

**Question 2. For whom did you primarily work, since the beginning of the pandemic?**

- National public health agency (for example, Public Health England/UK Health Security Agency, Public Health Wales, Public Health Scotland, Public Health Agency N. Ireland)
- Office for Health Improvement and Disparities (UK)
- Local authority / local government
- University or academic institution
- NHS Trust or Health Board
- NHS England and NHS Improvement
- Department for Health and Social Care / other national government department
- Non-profit organisation/NGO
- Private practice
- Other (please specify)
- Not applicable

**Question 3. Which best describes your role, since the beginning of the pandemic?**

- Director of Public Health
- Consultant or Specialist in Public Health
- Public Health Academic
- Public Health Trainee / Specialty Registrar
- Public health manager
- Public health nurse or midwife
- Public Health Practitioner
- Retired
- Other (please specify)

**Question 4. How long have you been working in a professional public health role?**

- 5 years or less
- 6 to 10 years
- 11 to 15 years
- 16 to 20 years
- More than 20 years

**Question 5. Are you clinically trained?**

- Yes
- No

**Question 6. Since the start of the COVID-19 pandemic, in your professional work, have you had to do something that you thought was ethically problematic (morally wrong) – and experienced distress because of it?**

**For example, you may have been required to say or do something because of a law or policy that you thought was incorrect, and this caused you distress.**

- Yes
- No / not sure

You said that in the last year, in your professional work, you had to do something that you thought was ethically problematic (morally wrong) – and experienced distress because of it.

For example, you may have been required to say or do something because of a law or policy that you thought was incorrect, and this caused you distress.

**7. How often did you experience this kind of situation?**

- 1 – Rarely
- 2
- 3
- 4
- 5 - Very frequently

**Question 8. Did this occur more or less frequently than in the 12 months before the pandemic?**

- Less often
- About the same
- More often
- Not sure
- I was not working in public health during that period

Now, think about the single incident of this type that caused you the most distress...

**Question 9. Please indicate the level of distress you experienced because of this situation**

- 1. A little
- 2
- 3
- 4
- 5. Great extent

**Question 10. How long did you continue to experience distress because of this situation?**

- Less than a day
- Between a day and a week
- Between a week and month
- More than a month

**Question 11. Did the distress you experienced cause you to take time off from work, and/or seek therapeutic help?**

- Yes
- No

**Question 12. Please can you briefly describe the situation that caused you distress? Please avoid using real names or details of specific workplaces etc.**

**Question 13. What would help avoid or mitigate situations like this?**

**Question 14. Since the start of the COVID-19 pandemic, in your professional work, have you done something that you thought was the ethical (morally right) thing – and experienced distress because of it?**

**For example, you may have made a decision that you knew was right, but someone criticised you for it, or it made life more difficult for someone, and this caused you distress.**

- Yes
- No / not sure

You said that since the start of the COVID-19 pandemic, in your professional work, you did something that you thought was the ethical (morally right) thing – and experienced distress because of it.

For example, you may have made a decision that you knew was right, but someone criticised you for it, or it made life more difficult for someone, and this caused you distress.

**Question 15. How often did you experience this kind of situation?**

- 1 – Rarely
- 2
- 3
- 4
- 5 - Very frequently

**Question 16. Did this occur more or less frequently than in the 12 months before the pandemic?**

- Less often
- About the same
- More often
- Not sure
- I was not working in public health during that period

Now, think about the single incident of this type that caused you the most distress...

**Question 17. Please indicate the level of distress you experienced because of this situation**

- 1. A little
- 2
- 3
- 4
- 5. Great extent

**Question 18. How long did you continue to experience distress because of this situation?**

- Less than a day
- Between a day and a week
- Between a week and month
- More than a month

**Question 19. Did the distress you experienced cause you to take time off from work, and/or seek therapeutic help?**

- Yes
- No

**Question 20. Please can you briefly describe the situation that caused you distress? Please avoid using real names or details of specific workplaces etc.**

**Question 21. What would help avoid or mitigate situations like this?**

**Question 22. Since the start of the COVID-19 pandemic, in your professional work, have you had to do something where you were not sure what the ethical (morally right) thing to do was – and experienced distress because of it?**

**For example, you may have been unsure who to prioritise for a service, or whether or not to recommend a particular course of action, and this caused you distress.**

- Yes
- No / not sure

You said that, since the start of the COVID-19 pandemic, in your professional work, you had to do something where you were not sure what the ethical (or morally right) right thing to do was – and experienced distress because of it.

For example, you may have been unsure who to prioritise for a service, or whether or not to recommend a particular course of action, and this caused you distress.

**Question 23. How often did you experience this kind of situation?**

- 1 – Rarely
- 2
- 3
- 4
- 5 - Very frequently

**Question 24. Did this occur more or less frequently than in the 12 months before the pandemic?**

- Less often
- About the same
- More often
- Not sure
- I was not working in public health during that period

Now, think about the single incident of this type that caused you the most distress...

**Question 25. Please indicate the level of distress you experienced because of this situation**

- 1. A little
- 2
- 3
- 4
- 5. Great extent

**Question 26. How long did you continue to experience distress because of this situation?**

- Less than a day
- Between a day and a week
- Between a week and month
- More than a month

**Question 27. Did the distress you experienced cause you to take time off from work, and/or seek therapeutic help?**

- Yes
- No

**Question 28. Please can you briefly describe the situation that caused you distress? Please avoid using real names or details of specific workplaces etc.**

**Question 29. What would help avoid or mitigate situations like this?**

**Question 30. Since the start of the COVID-19 pandemic, in your professional work, has your colleague(s), or organisation, done something that you thought was ethically problematic (morally wrong) – and you experienced distress because of it?**

**For example, a manager may have made a decision that you thought would cause harm or be unfair, and this caused you distress.**

- Yes
- No / not sure

You said that in the last year, in your professional work, your colleague(s), or organisation, did something that you thought was ethically problematic (morally wrong) – and you experienced distress because of it.

For example, a manager may have made a decision that you thought would cause harm or be unfair, and this caused you distress.

**Question 31. How often did you experience this kind of situation?**

- 1 – Rarely
- 2
- 3
- 4
- 5 - Very frequently

**Question 32. Did this occur more or less frequently than in the 12 months before the pandemic?**

- Less often
- About the same
- More often
- Not sure
- I was not working in public health during that period

Now, think about the single incident of this type that caused you the most distress...

**Question 33. Please indicate the level of distress you experienced because of this situation**

- 1. A little
- 2
- 3
- 4
- 5. Great extent

**Question 34. How long did you continue to experience distress because of this situation?**

- Less than a day
- Between a day and a week
- Between a week and month
- More than a month

**Question 35. Did the distress you experienced cause you to take time off from work, and/or seek therapeutic help?**

- Yes
- No

**Question 36. Please can you briefly describe the situation that caused you distress? Please avoid using real names or details of specific workplaces etc.**

**Question 37. What would help avoid or mitigate situations like this?**

**Question 38. Have you heard of the term ‘moral distress’ before?**

- Yes
- No
- Not sure

**Question 39. Have you heard of the term ‘moral injury’ before?**

- Yes
- No
- Not sure

**Question 40. Have you had any specific training in ethics during your formal public health education/training (e.g. as part of a BSc, MSc, MPH)?**

- No
- Not sure
- Yes (please specify)

**Question 41. Have you had any specific training in ethics after your formal public health education (e.g. a continuing professional development session on ethics)?**

- No
- Not sure
- Yes (please specify)

**Question 42. Do you believe that more training in ethics would have helped you handle the situation(s) you describe earlier?**

- Yes
- No
- Not sure
- Not applicable

**Question 43. Please indicate the extent to which each of these corresponds to your own situation?**

|  | 0 - Never | 1 | 2 | 3 | 4 - Always |
| --- | --- | --- | --- | --- | --- |
| I can easily determine if a situation contains an ethical/moral dilemma |  |  |  |  |  |
| I know what principles, tools or frameworks to use to help me make a decision when confronted with ethical/moral issues |  |  |  |  |  |
| I think I am adequately prepared to face the ethical/moral issues related to my practice |  |  |  |  |  |
| I think that my colleagues are adequately prepared to face the ethical/moral issues related to their practice |  |  |  |  |  |

**Question 44. What is your gender?**

- Female
- Male
- Other (please specify)
- Prefer not to say

**Question 45. What is your age?**

- Under 18
- 18-24
- 25-34
- 35-44
- 45-54
- 55-64
- 65+
- Prefer not to say

**Question 46. Do you consider yourself to have a disability?**

- Yes
- No
- Prefer not to say

**Question 47. What is your religion?**

- Christian – Church of England/Ireland/Wales/Scotland
- Christian – Catholic
- Christian – Orthodox
- Other Christian, please describe below
- Buddhist
- Hindu
- Jewish
- Muslim
- Sikh
- No religion
- Other, please describe below
- Prefer not to say

Please describe (if indicated above)

**Question 48. Which ethnicity best describes you?**

- White - Scottish/English/Welsh/Northern Irish/British
- White - Irish
- White - Gypsy or Irish Traveller
- White - Any other White background, please describe below
- Mixed/Multiple - White and Black Caribbean
- Mixed/Multiple - White and Black African
- Mixed/Multiple - White and Asian
- Mixed/Multiple - Any other Mixed/Multiple ethnic background, please describe below
- Asian - Indian
- Asian - Pakistani
- Asian - Bangladeshi
- Asian - Chinese
- Asian - Any other Asian background, please describe below
- Black - African
- Black - Caribbean
- Black - Any other Black/African/Caribbean background, please describe below
- Other - Arab
- Other - Any other ethnic group, please describe below
- Prefer not to say

Please describe (if indicated above)

**Question 49. Please leave your email address here if you would you be willing to discuss these issues in an interview or focus group:**

**Question 50. Do you have any further comments about this survey, and/or public health ethics in general?**
